# Factors affecting Accountable Care Organizations’ decisions to remain in or exit the Medicare Shared Savings Program following Pathways to Success

**DOI:** 10.1093/haschl/qxad093

**Published:** 2024-01-05

**Authors:** Meiling Ying, Jane H Forman, Sitara Murali, Lauren E Gauntlett, Sarah L Krein, Brent K Hollenbeck, John M Hollingsworth

**Affiliations:** Department of Foundations of Medicine, New York University Langone Health, New York, NY 11501, United States; Center for Clinical Management Research, Veterans Affairs Ann Arbor Healthcare System, Ann Arbor, MI 48105, United States; Department of Internal Medicine, University of Michigan Medical School, Ann Arbor, MI 48109, United States; Center for Clinical Management Research, Veterans Affairs Ann Arbor Healthcare System, Ann Arbor, MI 48105, United States; Center for Clinical Management Research, Veterans Affairs Ann Arbor Healthcare System, Ann Arbor, MI 48105, United States; Department of Internal Medicine, University of Michigan Medical School, Ann Arbor, MI 48109, United States; Department of Urology, Massachusetts General Hospital, Boston, MA 02114, United States; Department of Urology, NorthShore University HealthSystem, Chicago, IL 60201, United States

**Keywords:** Medicare, Pathways to Success, alternative payment models, qualitative methods

## Abstract

The Medicare Shared Savings Program (MSSP) is an alternative payment model launched in 2012, creating Accountable Care Organizations (ACOs) to improve quality and lower costs for Traditional Medicare patients. Most MSSP participants were expected to shift from bearing no financial risk to a 2-sided risk model (ie, bonus if spending reduced below historical benchmarks, penalty if not), yet fewer than 20% did. Therefore, in 2019, the Centers for Medicare and Medicaid Services launched the Pathways to Success program, which required shifting to a 2-sided model within 12 months. For the first time, more ACOs exited than entered the MSSP. To understand these participation decisions, we conducted qualitative interviews with ACO leaders. Pathways caused ACOs to reassess their potential shared savings vs losses, particularly in light of benchmarking methodology changes; reconsider perceived nonrevenue benefits; and reassess participation in the MSSP vs other programs. As ACOs, particularly those assuming downside risk, have contained costs and enhanced care quality, policymakers should strive to improve MSSP enrollment rates in downside-risk models through strategies that allow ACOs to achieve shared savings and deliver accountable care.

## Introduction

The Medicare Shared Savings Program (MSSP) is an alternative payment model (APM) launched in 2012, creating Accountable Care Organizations (ACOs) aimed at improving quality and lower costs for traditional fee-for-service Medicare beneficiaries.^1^ Participation in the program is voluntary, and entrants historically could select from 1 of 4 unique tracks with varying levels of financial risk and reward. Most chose Track 1 and bore no financial risk.^2^ Rather, participants were eligible for bonus payments if they reduced spending below historical benchmarks. Importantly, Track 1 participants were expected to shift toward a 2-sided risk model (ie, risk of penalty if spending benchmark was not met) as they matured, yet fewer than 1 in 5 did.^3,4^ Recognizing this, the Centers for Medicare and Medicaid Services (CMS) announced the Pathways to Success program in December 2018, consolidating the number of ACO tracks down to 2 and accelerating participating organizations’ transition to 2-sided risk in as little as a year.^5^

After the Pathways launch in 2019, more ACOs exited than entered the MSSP for the first time in the program's history. In 2020, 80 participants exited the program while only 53 joined.^6^ One explanation may be that exiting ACOs were simply not ready to bear the risk of having to repay millions if they were to fall short of spending benchmark targets.^7^ Alternatively, the magnitude of potential bonus payments in the 2-sided risk model may be insufficient to encourage continued participation. Regardless, decisions surrounding participation are complex and its calculus likely influenced by a host of factors, some of which may be shared across organizations. A better understanding of how ACO participation decisions were made in the context of Pathways implementation has important implications for the next generation of ACOs, as well a future Medicare payment policy.

We conducted a qualitative study, consisting of semi-structured interviews with ACO leaders, to understand factors influencing decisions to continue participation in or exit the MSSP after the Pathways launch. Further, among dropouts, we assessed planned participation in alternatives to the ACO payment model.

## Data and methods

### Study design and sample

We used a descriptive qualitative approach following a naturalistic philosophy, whereby something is studied in its natural state and findings reported in “data-near” terms,^8,9^ to better understand the factors that influenced the MSSP participation decision, as described by key stakeholders.

Using administrative data (CMS Accountable Care Organizations Public Use File),^10^ we identified Track 1 ACOs. We purposively selected organizations that were up for contract renewal in July 2019, coincident with the launch of Pathways, and distinguished between those that stayed in vs exited the MSSP. Through a combination of personal connections, advertisements in ACO newsletters, and email invitations to administrators at organizations in our sample frame, we found initial contacts at 9 ACOs who agreed to be interviewed. From these initial contacts, we used a snowball approach to identify other key organizational stakeholders with different roles (eg, chief executive officer, chief medical officer, executive director, director of quality and clinical operations) for recruitment at these ACOs. Individuals were invited to participate in study interviews via email. We offered a $400 honorarium as an incentive to individuals who participated in our 1-hour interview. This study was approved as exempt by the University of Michigan's Institutional Review Board.

### Data collection

We constructed the semi-structured interview guide using a conceptual model that draws on theories of health care organization and innovativeness.^11,12^ Our interview questions focused on general ACO organizational characteristics and strategic, organizational, and structural factors that influenced their participation decision. We pilot-tested the interview guide with ACO1 participant 1, who represented 1 of our key stakeholder groups, and refined our approach to recruitment and the guide accordingly.

Prior to the first interview at each ACO that stayed in the MSSP (*n* = 5), we conducted a brief survey focused on organizational structure. The survey included questions about organizational leadership type; size in terms of attributed beneficiaries, number of component facilities (eg, hospitals, skilled nursing facilities, acute rehabilitation facilities), providers, and provider groups (eg, specialty physician groups, primary care physician groups); participation in other risk-based contracts; and contact information for members of the leadership board. Answers from the survey were used for recruitment and in preparation for interviews.

We conducted semi-structured interviews between January 2021 and January 2022. We conducted all interviews with video communication software. Two or more members of the study team (principal investigator, co-investigator, qualitative researcher, qualitative analyst, research coordinator) participated in each interview. The average interview length was 52 minutes (range: 29–74). We recorded and transcribed verbatim all interviews.

### Data analysis

We conducted qualitative content analysis using a combination of deductive and inductive approaches. We developed a summary template to organize individual interview data by strategic, organizational, and structural factors represented in the interview guide. The lead interviewer and notetaker(s) completed a brief summary following each interview. Next, members of our study team created in-depth summaries for each organization, organizing data from all interviews at each site according to the organizing framework. We identified inductively elements not captured in the initial organizing framework and added them to the organizational summaries. A second analyst reviewed each summary, and we resolved discrepancies through discussion. We used these final summaries to populate a matrix containing all sites, which allowed us to examine patterns across sites, and develop findings.

### Limitations

Our study must be considered in the context of limitations. First, we were unable to determine ACO type for all organizations in our sample frame and are only able to provide a detailed description for participating ACOs (*n* = 9). Second, this study may not have captured all factors relevant to decisions about participation in the MSSP program. Although we purposely included ACOs encompassing academic and nonacademic medical centers, and varied geographic locations, our sample overrepresented physician-hospital–led ACOs and included no hospital-led ACOs. Third, it is important to note that our study is qualitative, semi-structured, interview-based research and is not intended to be generalizable. However, our findings may be transferable to similarly situated health systems.^13^ Finally, an additional challenge in investigating ACO MSSP participation is that there are apparent variations in response to our interview invitations, which could potentially bias our results. During the participant recruitment period, we experienced greater challenges in contacting ACOs that exited the MSSP after Pathways. Moreover, during the interview, ACOs that remained in MSSP might be more willing to discuss their decision making than ACOs that exited. To address these potential sources of bias, we took multiple measures: ensuring confidentiality and anonymity in our data-collection process, conducting pilot testing with a small group of participants, offering financial incentives to maximize response rates, involving multiple individuals in each interview to communicate the purpose and instructions clearly, utilizing multiple analysts for data analysis, and emphasizing the importance of candid and honest responses from all participants.^14^

## Results

Nineteen clinicians, executives, and administrators at 9 ACOs participated in our study (Table 1). The majority of ACOs were physician–hospital partnerships with an academic medical center affiliation. The ACOs were located in the Midwest, Southwest, and mid-Atlantic United States.

**Table 1.** Study participants.

	ACO type	Geographic region	Interviewee roles	Pathways participation
ACO1	Physician–hospital partnership AMC affiliation	Midwest	Executive Director, ACO1 and PO1 Executive Director, PO2 Executive Director, PO3 Clinical Operations Director, PO3 Chief Clinical Integration Officer, PO4 Physician Group President, PO4	Yes
ACO2	Physician–hospital partnership	Midwest	Chief Medical Officer Chief Operations Officer Director of Care Coordination	Yes
ACO4	Physician–hospital partnership AMC affiliation	Southwest	Director of Government Programs & Post-Acute Care	Yes
ACO5	Physician–hospital partnership	Midwest	Vice President of Government and Value-Based Programs Vice President of Population Health	Yes
ACO6	Physician-led	Midwest	Vice President of Population Health Director of Quality Operations	No
ACO7	Physician–hospital partnership AMC affiliation	Midwest	Assistant Vice President for Health and External Affairs	No
ACO8	Physician-led AMC affiliation	Midwest	Medical Director Vice President of Institute for Quality, Innovation and Patient Safety	No
ACO9	Physician–hospital partnership AMC affiliation	Mid-Atlantic	Executive Vice President, Insurance	No
ACO10	Physician–hospital partnership AMC affiliation	Mid-Atlantic	Senior Director of Quality and Value-Based Care	No

Source: Author collected data.

Abbreviations: ACO, Accountable Care Organization; AMC, Academic Medical Center; PO, Physician Organization that is an ACO member.

We identified 4 themes that capture the main factors reported by interviewees, which affected their organizations’ decision making around MSSP participation after launch of Pathways to Success: (1) the perceived nonrevenue benefit to the organization from the program, (2) the importance of the program as a revenue source, (3) the effect of benchmarking changes on the potential for shared savings, and (4) the relative advantage of participating in the program vs others. These themes are discussed in detail below with additional supporting quotes provided in Table 2.

**Table 2.** Additional supporting quotations.

	Quotations
Perceived nonrevenue benefit to the organization	
Stayed	“We also have a decent bit of just older population that haven't really bought into the Medicare Advantage plans just yet. They haven't seen the advantage of them. So, you know, it's honestly a good feeder system into Medicare Advantage.”—ACO6 “And so the opportunity to partner with [ACO1 PO1] came up and it felt like a really good partnership. They have a depth of resources that we don’t have, including data analytics.”—ACO1 PO3 “The [ACO1] board then has an opportunity to set aside the shared savings into our shared risk pool, which is one of the structures that we use to bear down-side risk…these are the moneys set aside in the event we have a shared loss.”—ACO1 “What we are really trying to do is this is the way we should just treat all patients vs [just] our value-based care patients…in a Medicare type of setting…it is starting to be that when a physician sees one patient, they see they are a Medicare patient, that they can start to understand that especially quality of care is the way we treat all of these patients at this age which, quite honestly, is a helpful thing…Being able to participate in a program like this that has such large numbers has allowed for the program to be so visible.”—ACO5 “What is great is that [the MSSP] population aligns really well with our Medicare Advantage population in terms of general patient needs and how to care for them. And so, what we try to do from the [ACO] side is if we create an initiative, try to make sure it's kind of a global initiative that will easily benefit all of our payer populations instead of really segmenting care for a particular population.”—ACO6
Importance of MSSP as a revenue source	
Stayed	“So, we will have to brag a little bit just in our MSSP savings. So, for 2019 and ’18, we have savings—’19 is shown here so that's 68 million total and then we were able to bring 34 of that back into the system.”—ACO4 “I would say [MSSP is] a huge driver in the sense that the last several years we have been able to see a significant amount of dollars come through this program.”—ACO5 “We have had a pretty good streak. I think we had shared savings since 2018 performance year.”—ACO6
Exited	“It's very hard to earn shared savings in MSSP and we had not earned any savings.”—ACO7 “MSSP for both rounds, we didn't get a bonus payment. So, the first round, I think, we were short by like one percent. I don't know, it was darn close, and we didn't see any bonus payment. And then the last round, equally, we were just so close.”—ACO8
Effect of benchmarking changes on the potential for shared savings	
Stayed	“We work with actuarial teams to evaluate our risk, and so we definitely go into it understanding what our maximum gain and loss could be, and our likely scenarios would be, but I would say there wasn’t a lot of debate on it if we should do it.”—ACO4
Exited	“We overinvested in things like analytics at a time that we couldn’t recover return fast enough out of MSSP when our benchmarks were some of the lowest in the United States … because we have the lowest Medicare reimbursement in the United States, and we have high quality and low cost, so our total cost of care spend is just low.”—ACO7 “We had taken [out] a fair amount of the waste…and all of the ground we had gained now became the line in the sand that we were measured against. I always kind of likened it to a hamster wheel…you run fast on the wheel but the faster you go, the faster you have to run in order to continue to make headway…the chance of us removing a significant amount of cost from the system to…clear the threshold that was set and have enough coming back to us to support the work and infrastructure to do that became a non-sustainable model.”—ACO8
Relative advantage of participating in the MSSP over others	
Stayed	“Some of the decision making behind taking on the downside risk with MSSP had to do with MIPS, obviously given the five percent uptick as an alternative payment model. I think that was one of the drivers to go ahead and proceed with the downside risk.”—ACO1 PO2 “We could never generate enough revenue [from fee-for-service medicine] to really grow the support around the patients that we wanted to grow. …So, we welcomed the challenge, in a way, to start moving down that path.”—ACO6 “We would rather be in MSSP vs MIPS or anything like that, for sure. … MIPS…it's just easier for us reporting-wise.”—ACO6
Exited	“We still had [COMPETING PROGRAM] which [is]…structurally not too far different [from MSSP], less opportunity for gains share, but on the flipside, more upfront money because of the per member per month payments we get for doing care coordination… it just didn't make sense for us to re-enter MSSP.”—ACO9 “We struggled to really bend the cost curve on our hospital expense, and part of that…was based on the [ACO10 STATE] model in the way hospitals have global budgets…you may be cutting out utilization and you may be cutting out costs but because of the way the global budget works, your charges, right, are not accounting for that.”—ACO10

Source: Authors’ analysis of data collected during semi-structured interviews.

Abbreviations: ACO, Accountable Care Organization; MIPS, Merit-based Incentive Payment System; MSSP, Medicare Shared Savings Program; PO, physician organization.

### The perceived nonrevenue benefit to the organization from the program

One of the main factors influencing an ACO's decision around MSSP participation after Pathways’ launch is the perceived nonrevenue benefit to the organization. For ACO1, which is a confederation of physician organizations (POs) and hospitals in a single state, the MSSP is its *raison d’etre*. Further, 1 interviewee from ACO1 described the commitment of 1 of its constituent POs to providing access to Medicare fee-for-service beneficiaries, in the face of growing market pressures to cater to Medicare Advantage plans:

“Ask … why involve yourself in a large [traditional] Medicare population and not just make a heavy push into Medicare Advantage and try to minimize your fee-for-service [beneficiaries]. And I would tell you that, culturally, that will never fly in [ACO1 PO1] … [ACO1 PO1 has] a commitment to being available to the citizens—the people of the state. There will always be a [traditional] Medicare population that will need access to [ACO1 PO1], and it will never be a trivial [number].”—ACO1

In addition, ACO1 and its constituent POs appreciated access to data and analytics and the opportunity to learn from each other in a *de facto* quality and learning collaborative model.

“I mean what an amazing learning culture. And it really is a learning culture because we do okay with what we have, but what we have been able to learn because of our participation in [ACO1] is remarkable … there is value to hearing what the team is doing in [other ACO1 POs]…that helps us to improve .… A lot of what we talk about is turning data into action and how have we been able to make it intervenable within [our] community … everything we talk about … particularly the operations side, is how you implement things … . And those are things we are better because of it.” —ACO1 PO2

For ACO2, the MSSP was used strategically to help engage their physician members in other risk-based contracts. ACO6 had a similar motivation, seeing MSSP as a “good feeder system into Medicare Advantage.”

“We are actually engaging [physicians participating in the MSSP] in other [risk-based contracts] … piggybacking on MSSP with a little risk, but then we are attracting them to sign up [for others] through us … [So,]... as MSSP shrinks the carrot, we are increasing other parts to more than compensate for that.”—ACO2

### The importance of the program as a revenue source

A second factor influencing an ACO's decision around MSSP participation after Pathways’ launch is the program's importance as a source of revenue for the organization. Namely, for 5 of the 6 ACOs with which we spoke that maintained participation, shared savings payments from the MSSP contributed greatly to their bottom line.

“We have 14 contracts but … MSSP is our biggest one and our livelihood … [it] has driven most of our success.”—ACO2

ACOs 4, 5, and 6 also described the importance of MSSP shared savings payments as supporting the provision of services they valued highly. Their care management programs were funded, in part, by these payments, which allowed them to provide better care for all their patients, regardless of payer.

“What we are doing … [is] more [broad] than just MSSP … what we are trying [not to] do is make our care model or interventions specific to … [a Medicare Advantage] population or MSSP or bundles … because it's hard for the providers to understand what is happening, so we are trying to make it more streamlined, this is what we provide you, and less about each contract.”—ACO4

Among those organizations that maintained their MSSP participation, only ACO6 prioritized the MSSP lower on the revenue scale than the full-risk plans in which it engaged like Medicare Advantage. Although ACO6 had a large number of beneficiaries enrolled in traditional Medicare assigned to it, shared savings payments from MSSP participation were capped. But no such cap existed for their full-risk plans, which made those plans a higher organizational priority.

“[In the] MSSP, … we are at [the] max 50% sharing rate … [whereas] for our full risk populations, … it's a 100% sharing rate. So, that's kind of the difference with returns in terms of patient volume … If we had to prioritize our efforts—because you have to, that's reality, right, when you have limited resources—[the MSSP is] not the number one priority per se. But they are up there for sure.”—ACO6

In contrast to the ACOs that maintained their participation in the MSSP, ACOs 7 and 8, which left the MSSP, had eligible beneficiary populations that weren’t large enough to be able to earn a significant amount of shared savings. These ACOs had more diverse payer mixes, in which Medicare fee-for-service beneficiaries accounted for a relatively small proportion of the overall population served. Consequently, these ACOs earned little to no shared savings during their time in the program, motivating their decision to leave it, along with their assessments of potential savings under Pathways. On the other hand, ACOs 9 and 10, which also chose to leave the MSSP, had done well on shared savings, but other factors that we describe below drove their decision to end MSSP participation.

### The effect of benchmarking changes on the potential for shared savings

When faced with taking on downside risk under Pathways, organizations that we interviewed determined their potential for earning shared savings (vs losses). These potentials shifted after launch of Pathways due to changes that CMS made to the MSSP benchmarking methodology. Under the ACO's initial contract, its benchmark was based on its own historical spending during a baseline period, trended forward at the national rate of Medicare spending growth. Because this approach created unequal benchmarks for ACOs in the same market, CMS introduced a regional adjustment when an ACO renewed its contract that effectively blended its historical spending with the average spending in its region. Pathways hastened the transition to such regionalized benchmarking, which was advantageous for some organizations, like ACO6, whose historical benchmark was significantly below its regional benchmark.

“For the first couple years, we definitely did not have shared savings. We also started with the MSSP program … with a pretty low benchmark, and so it since kind of normalized with the regional way that they look at benchmarks … .So, that has helped us to kind of get a better right-sized benchmark so that we can share in savings.”—ACO6

For ACO1, which opted to continue participating in the MSSP, its benchmark actually fell with the blending of historical and regional spending trends. Despite this, organizational leaders thought that there was still enough room for them to achieve shared savings because they believed that the organization would realize efficiency gains above national Medicare fee-for-service levels.

“I think, historically, a lot of our shared savings … has been a little bit an arbitrage between the national increase in expenditures in the way the benchmarks were calculated and our local performance. Some of it is real, actual performance. I can point to readmission decreases, over all hospital admission decreases, general decreases in utilization across our patient population … some of it is arbitrage from national increases in fee-for-service spending baked into our benchmark when regional increases were smaller.”—ACO1

That said, ACO1 had a high market share in its region. Although CMS tried to account for this issue, weighting national trends by an ACO's average market share, some interviewees at ACO1 still expressed anxiety around it.

“The more they move to regional benchmarks, the more I am competing against myself. So, [given my market share] … you are going to compare me to myself, [and] I’m going to lose 100% of the time.”—ACO1

In contrast, ACO7 and ACO8, which left the MSSP after Pathways, had already-low benchmarks even before CMS's methodology change and were struggling to generate enough shared savings to cover their infrastructure costs. ACO8 said that it did not make sense to take on downside risk in those circumstances.

“with the [existing] benchmarking issue that we had, and all of these other pieces, taking on additional risk for very little upside potential just didn’t seem to make financial sense to us as an organization.”—ACO8

Finally, an interviewee from ACO8 summed up what they saw as flaws in benchmarking policy generally.

“There's got to be this inflection point, right, where for a population, there's a magical number that if you spent just this amount on average for managing your population, you would maximize your quality and outcomes, and minimize your costs over time. And we should be rewarding organizations who maintain a presence at or near that inflection point, in a longitudinal way …. You have to build an infrastructure and maintain things that keep you at that point, but if the revenues go away every time you re-benchmark, which you would under the way that this is traditionally run, you know now, you have no way to maintain that infrastructure.”—ACO8

### The relative advantage of participating in the program over others

In discussing their ACO's MSSP participation decision, several interviewees described the relative advantage of participating in the program over other CMS-approved advanced APMs like the Comprehensive Primary Care Plus Model and the Next Generation ACO Model or the Merit-based Incentive Payment System (MIPS)—a new payment track created under the Medicare Access and CHIP Reauthorization Act that builds on traditional fee-for-service payments by adjusting them up or down based on a physician’s or physician practice's performance in a new reporting system. For instance, ACO5 was motivated, in part, by an incentive payment that the organization would receive for performance years 2017 to 2022 by continuing on and accepting downside risk in the MSSP after launch of Pathways.

“What I think affected our decision the most was actually … providing the advanced APM .… We were in that period where they allowed you to switch if you were on a Track One …. So, we did that, and that was … based on being able to get that five percent … bonus.”—ACO5

Interviewees from ACO5 also mentioned that there was a dearth of risk-based contracting alternatives to MIPS in 1 of the states where their organization is located.

“Especially in our [ACO5 STATE2] market, we don’t have a very big penetration of [risk-based contracts], and so this is a great opportunity for us to continue with value-based care … [the MSSP] provides the support and the structure that, sometimes, traditional fee-for-service does not provide; that, I truly believe is good care for patients.”—ACO5

Three ACOs that exited the MSSP did so in favor of other advanced APMs. ACO7, based out of an academic medical center, faced challenges in the MSSP because it was responsible for providing a large proportion of specialty care to the majority rural population that it serves. Therefore, it joined a larger Next Generation (NG) ACO to avoid additional infrastructure investment and to dilute its risk. Although the NG ACO had higher levels of risk than the MSSP, the NG ACO offered ACO7 some protection from downside risk.

“Being an academic medical center, we had at this point not chosen to make kind of a higher level investment infrastructure that you would want to have in order to feel comfortable taking that level of downside risk, and so the opportunity of being part of a larger NextGen [ACO] that's very high performing has been an attractive option for us because we get sort of the benefits of [the] NextGen [model] but by being a sub-contractor, the way their ACO is structured, we do have some protection from that downside risk.”—ACO7

ACOs 9 and 10 are located in a state that had partnered with the CMS Innovation Center to modernize the state's all-payer rate-setting system for hospital services in an effort to improve health and reduce costs. Under this initiative, each hospital in the state receives a population-based payment amount to cover all hospital services provided during the course of the year, and hospitals can make incentive payments to nonhospital providers who partner and collaborate with the hospital and perform care redesign activities. The promise of these payments made the potential of shared savings through MSSP participation less appealing.

“The [COMPETING PROGRAM], you know, I mean it kind of was a no-brainer … there was a care management fee so there was a reliable source of revenue that allowed you to invest in the practices … frankly, we were not alone. Most ACOs in the state of [ACO10 STATE] removed themselves [from MSSP].”—ACO10

ACO8 was the only ACO in our study to exit the MSSP in favor of MIPS. Interviewees stated that MSSP reporting requirements were too onerous, and the organization frequently had to divert care team members from their normal work to help with it.

“[With the MSSP] almost every year, the reporting tools aren’t working correctly … you are having a challenge uploading your data, and you call. And you can’t get an answer, and it's very, very resource intensive .… We literally pulled our quality nurses almost 100% offline for [2 months] to get all of our reporting done .… The opportunity costs of not having … [nurses involved with] improvement efforts… but instead, stuck in an office auditing charts was a huge investment of time.”—ACO8

Moreover, interviewees from ACO8 liked having choice in the measures that the organization reported under MIPS.

“[With] MIPS, when we get onto a certified medical record, we can pick the measures, whereas with the MSSP, we really couldn’t. And this is the deal, these are the measures that you have. So, we didn’t have that flexibility.”—ACO8

## Discussion

The launch of Pathways to Success represented the first major overhaul to the MSSP since inception, requiring participants to shift to a 2-sided risk model within 12 months. Our national qualitative study of 21 clinician leaders, executives, and administrators at 9 ACOs revealed that changes ushered in by Pathways caused ACOs to reassess their individual calculus for potential shared savings vs losses. Further, the policy prompted leaders to reconsider any perceived nonrevenue benefits associated with the MSSP (eg, opportunities to promote care access and quality for all patients, and to acquire experiences from other participants) and reassess the pros and cons of continued participation as opposed to entry into other alternative advanced payment models or the MIPS. For some, the calculus was favorable and supported continued participation. Not so for others.

Nonrevenue benefits of the MSSP described in interviews included the following: understanding the shift from a focus on beneficiary volume to value-based care and collaboration among provider and hospital organizations within ACOs. Although participation in value-based payment models began with joining the MSSP, most MSSP participants have subsequently engaged in additional contracts linking payment, at least partially, with quality through other federal programs or the private sector.^15,16^ From the interviews, it was clear that there was broad recognition that the business model of focusing solely on increasing patient volume was not viable. To be successful in the 21st century, organizations would need to be proficient at demonstrating value, through improving clinical outcomes, enhancing care coordination, or eliminating waste. ACO leaders also described the benefits of collaboration across organizations and access to data and analytics.

The organizational calculus around continued participation was strongly influenced by the following 2 factors: (1) proportion of overall population served that was accounted for by Medicare fee-for-service beneficiaries and (2) the participant's historical spending benchmark. For the former, participants with a more diverse payer mix did not feel that the financial upside under Pathways was great enough to take on downside risk, leading them to exit. For the latter, participants with a lower historical spending benchmark prior to Pathways (suggesting perhaps already efficient health care delivery and making further improvements more difficult) were at a disadvantage in terms of achieving bonus payments, leading to a higher likelihood of dropping out.^17^ With Pathways implementation, all ACOs necessarily took a close look at the new benchmarking methodology before agreeing to take on downside risk. All interviewed participants made major investments in population health and care management programs to help realize value for those that they served. Nonetheless, in the 2-sided risk model, such investment may not only go completely unrecognized (ie, no bonus payment) but even be punished through penalty, if the spending benchmarks to meet were unfavorable.

Our findings have important policy implications. Due in part to the revised benchmarking methodology described above, CMS has seen a plateau in the number of beneficiaries assigned to MSSP ACOs. To the extent that care receipt benefits Medicare beneficiary health, this trend could worsen existing health inequalities. This is likely because Pathways might affect a small part of an MSSP ACO's population. Following Pathways, higher spending populations and racial/ethnic minorities are increasingly underrepresented in the program, such that non-White and dual-eligible beneficiaries are less likely to be assigned to an MSSP ACO than their counterparts.^6^ Moreover, the exodus of ACOs from the MSSP after Pathways could result in an unanticipated massive financial loss for Medicare. Prior empirical work estimates that potential savings lost due to exits could reach nearly $400 million for the most recent fiscal year.^18^ Our findings would suggest that some of the recently announced adjustments to the benchmarks embedded in the MSSP (eg, changes to regional adjustment by restricting the cap of the negative regional adjustment and accounting for an ACO's percentage of dually eligible beneficiaries and Hierarchical Condition Category risk score change, amending the risk score cap for the current benchmarking algorithm to consider changes in severity and case mix within an ACO's assigned population, benchmark adjustment to account for prior ACO savings)^19^ could help reverse these concerning inequity and disenrollment trends. In addition, other changes to the MSSP, like providing financial support to new entrants, could also mitigate these concerns.^19-21^

## Conclusion

Following the implementation of Pathways to Success, an ACO's decision around MSSP participation was primarily driven by 4 factors: the potential of future shared savings, perceived nonrevenue benefits from the MSSP, benchmarking policy, and comparative advantages offered by the MSSP compared to other initiatives. ACOs, particularly those assuming downside risk,^4^ have demonstrated the ability to contain costs and enhance care quality.^22,23^ Therefore, policymakers should strive to improve ACOs’ MSSP enrollment rates in downside-risk models by supporting or optimizing strategies that allow ACOs to achieve shared savings while generating cost savings to the government and care improvement to beneficiaries.

## Supplementary material

Supplementary material is available at *Health Affairs Scholar* online.

## Supplementary Material

qxad093_Supplementary_Data
